# Extraskeletal osteosarcoma of the thorax in a goat: case report

**DOI:** 10.1186/1746-6148-7-55

**Published:** 2011-09-19

**Authors:** Ueli Braun, Colin C Schwarzwald, Eva Forster, Mareike Becker-Birck, Nicole Borel, Stefanie Ohlerth

**Affiliations:** 1 Department of Farm Animals, Vetsuisse Faculty, University of Zurich, Zurich, Switzerland; 2 Department of Horses, Vetsuisse Faculty, University of Zurich, Zurich, Switzerland; 3 Institute of Veterinary Pathology, Vetsuisse Faculty, University of Zurich, Zurich, Switzerland; 4 Diagnostic Imaging Section, University of Zurich, Zurich, Switzerland

## Abstract

**Background:**

This report describes the results of clinical, ultrasonographic and computed tomographic examination of a 16-year-old goat with extraskeletal osteosarcoma of the thorax.

**Case presentation:**

The lead clinical signs were abnormal condition and demeanour, fever, tachycardia, tachypnoea, dyspnoea and dilated jugular veins. Ultrasonographic examination of the thorax revealed a precardial mass, measuring 16.4 by 11.4 by 14.2 cm. Computed tomographic examination showed dorsocaudal displacement of the trachea, heart and lungs to the right. A tentative diagnosis of mediastinal or pleural neoplasia was made, and the goat was euthanased and necropsied. A definitive diagnosis was based on histological examination of the mass.

**Conclusions:**

To our knowledge, this case report is the first description of extraskeletal osteosarcoma of the thorax in goats and serves to broaden the diagnostic spectrum of thoracic diseases in this species. Extraskeletal osteosarcoma should be part of the differential diagnosis in goats with thoracic tumours.

## Background

Tumours of the thorax are rare in goats [1]. Thymoma is the most common thoracic neoplasia in this species [2-6] and there are a few reports of lymphosarcoma and ovine pulmonary adenocarcinoma [1]. To the authors' knowledge, osteosarcoma has not been described in goats. Osteosarcoma occurs most commonly in the long bones and is rare in domestic animals other than cats and dogs [7]. Osteosarcoma may occur in organs other than the skeletal system, and the absence of a primary bone lesion is a prerequisite for the diagnosis of extraskeletal osteosarcoma. In dogs, extraskeletal osteosarcoma has been reported in the spleen, gastrointestinal tract, urogenital tract, liver, skin, mammary gland and subcutis [8-10]. A dog with osteosarcoma of the os penis was recently described [11]. Osteosarcoma commonly metastasizes in dogs, and haematogenous metastasis is more common than lymphogenous metastasis [9]. The major target organs include the lungs, liver, mediastinum, omentum and heart [8]. There have been two reports on osteosarcoma in goats; one case involved osteosarcoma of the humerus in a Toggenburg goat [12] and the other described osteosarcoma of the metacarpus of a seven-year-old Alpine goat [13]. The purpose of this report was to describe the clinical, ultrasonographic and computed tomographic findings in a goat with extraskeletal osteosarcoma.

## Case presentation

A 16-year-old castrated male miniature goat was referred to our clinic because of poor appetite, tachypnoea and tachycardia of several weeks duration. The general condition and demeanour were abnormal, the rectal temperature was 40.2°C (normal, 38.1- 40.0°C, [14]), the heart rate was 160 beats per minute (normal, 70 - 90 beats per minute, [14]) and there was a mild arrhythmia. Both jugular veins were distended. The goat had dyspnoea, abdominal respiratory effort and a respiratory rate of 60 breaths per minute (normal, 15 - 30 breaths per minute, [14]). Auscultation of the lungs revealed increased respiratory sounds. Examination of the intestinal and urinary tracts, musculoskeletal system and central nervous system yielded no abnormal findings.

Based on reference values established in this clinic [15], the activities of glutamate dehydrogenase (103 U/l, normal 3.1 - 19.8 U/l) and sorbitol dehydrogenase (128 U/l, normal 20.4 - 68.7 U/l) were increased and the serum concentrations of magnesium (0.64 mmol/l, normal 0.9 - 1.4 mmol/l) and inorganic phosphate (0.85 mmol/l, normal 1.2 - 2.9 mmol/l) were decreased. Other biochemical variables and a complete blood cell count were within normal limits.

**Figure 1:**
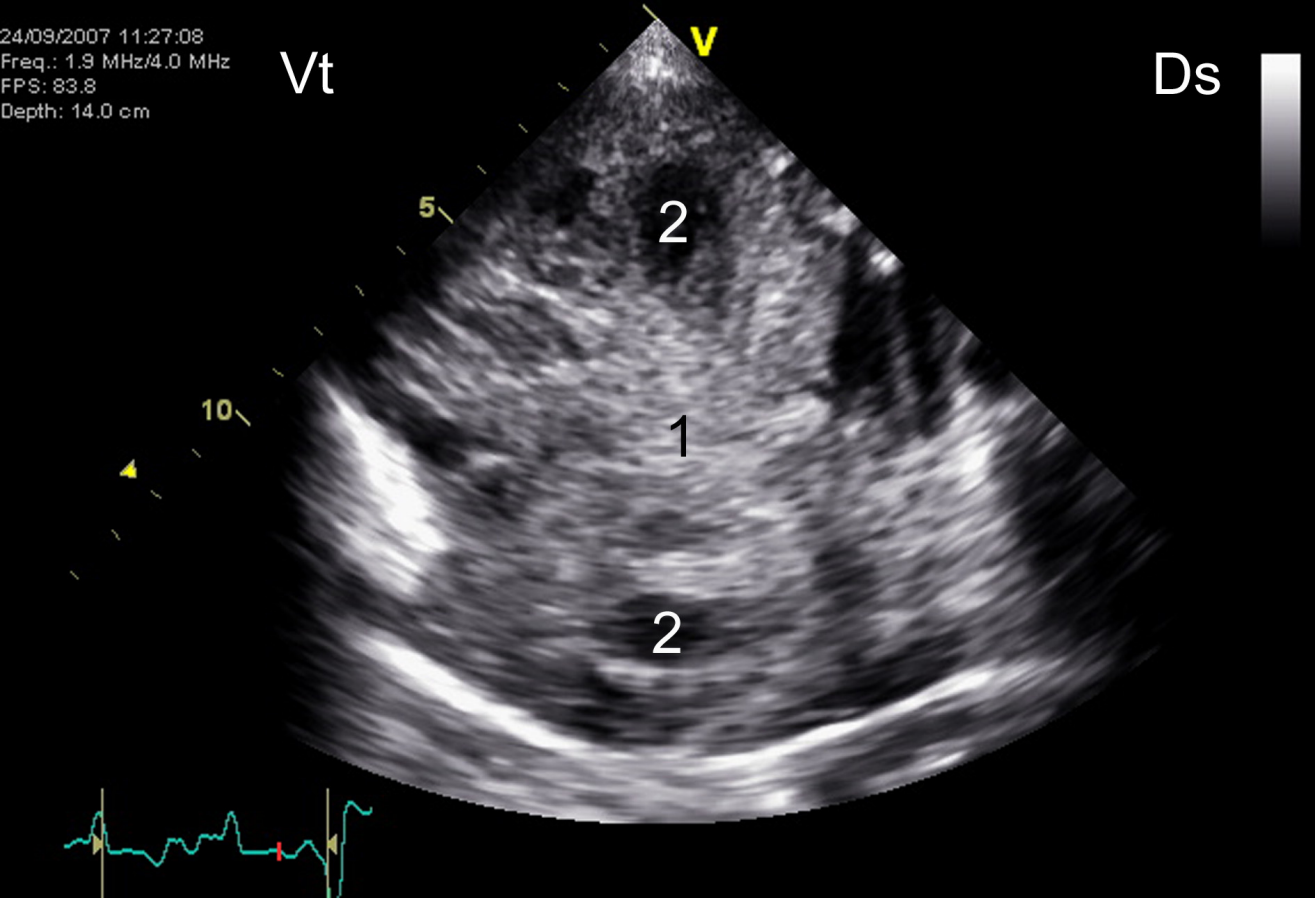
**Ultrasonogram of osteosarcoma**. Ultrasonographic appearance of a thoracic precardial mass in a miniature goat. The mass was round and multicystic and had a diameter of approximately 11 cm. A sector scanner with a phased-array probe and a frequency of 1.9 to 4.0 MHz (octave harmonics) was used. 1 Mass, 2 Cyst, Ds Dorsal, Vt Ventral.

Ultrasonographic examination of the thorax and heart using a phased-array sector probe (1.9/4.0 MHz octave harmonics, GE Vivid 7 Dimension, GE Medical Systems, Glattbrugg, Switzerland) revealed a multi-chambered precardial mass measuring 11 cm in diameter (Figure 1) and mild pleural and pericardial effusion. Electrocardiography showed ventricular extrasystoles, indicating secondary lesions of the myocardium. Subjectively, the right ventricle appeared enlarged compared to the left ventricle, but no obvious structural changes of the heart were detected during echocardiographic examination.

**Figure 2:**
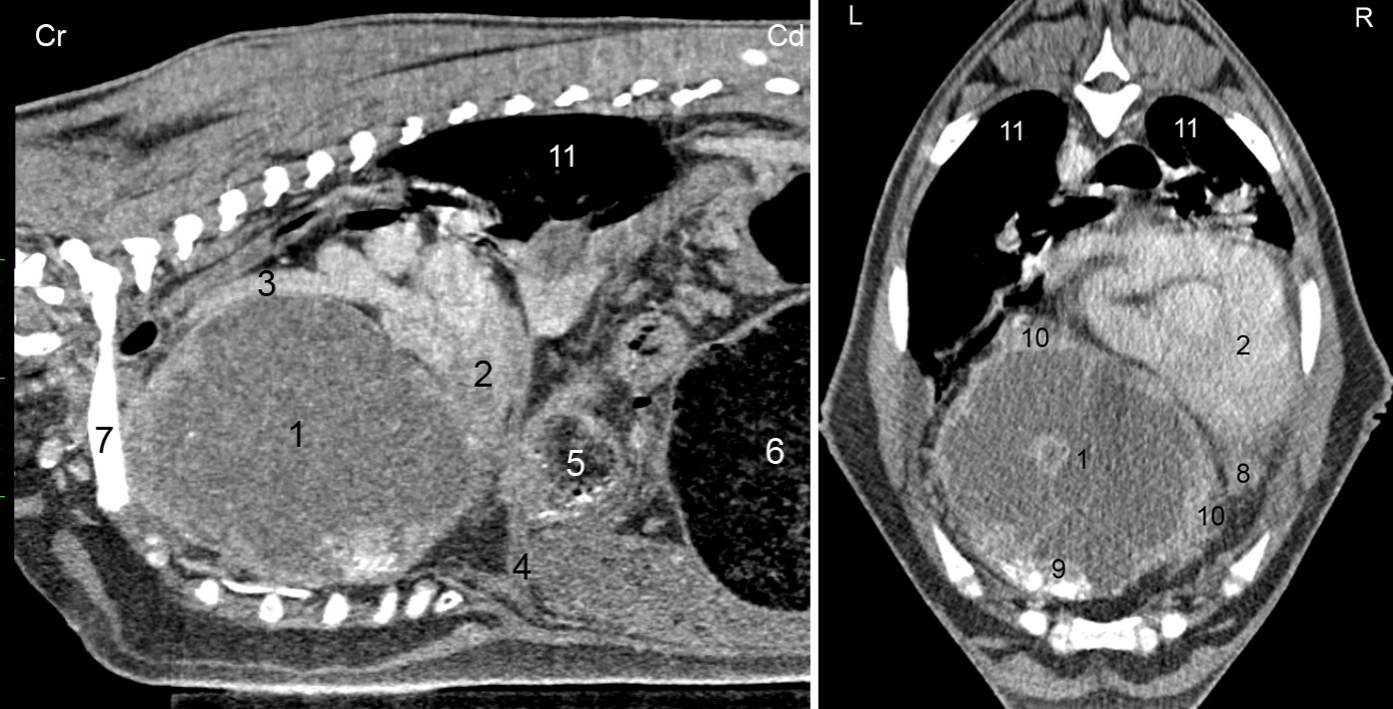
**CT image of thoracic osteosarcoma**. Computed tomographic soft-tissue window after intravenous application of contrast medium in a miniature goat with thoracic osteosarcoma. Sagittal section of the right hemithorax (A) and transverse section at the level of the 8th thoracic vertebra (B). The mass extended from the thoracic inlet to the diaphragm and displaced the heart dorsally and to the right. Accumulation of contrast medium was limited to the periphery of the mass. A smaller nodular mass was seen ventral to the heart. 1 Precardial mass, 2 Heart, 3 Truncus brachiocephalicus, 4 Diaphragm, 5 Reticulum, 6 Rumen, 7 First rib, 8 Nodular mass, 9 Amorphous mineralization, 10 Peripheral accumulation of contrast medium, 11 Lung, Cr Cranial, Cd Caudal, L Left, R Right.

A non-contrast computed tomographic (CT) examination of the thorax as well as CT angiography were carried out using a 40-slice CT scanner (Somatom Sensation Open, Siemens, Erlangen, Germany). General anaesthesia was induced and the goat was positioned in sternal recumbency and stabilized with multiple foam blocks. Transverse contiguous slices were obtained from the thoracic inlet to the cranial abdomen. Technical settings were 120 KV, 200 mA, 1 s tube rotation, 5 mm slice collimation and a pitch of 1.0 and 0.8 for the non-contrast scan and CT angiography, respectively. The data were reconstructed to an image series with 1.5 mm slice thickness using a medium-frequency image reconstruction algorithm (soft tissue) and a high-frequency image reconstruction algorithm (bone), respectively. CT angiography was carried out according to a recently published protocol [16]. CT images were transferred to a work station and reviewed with dedicated software (OsiriX Open Source™ Version 3.2.1, OsiriX Foundation, Geneva, Switzerland). The precardial mass, which was located on the ventral aspect of the thorax and measured 16.4 × 11.4 × 14.2 cm, extended from the thoracic inlet to the diaphragm (Figure 2). There was marked displacement of the heart and trachea dorsocaudally and to the right. The lungs and bronchi were compressed and displaced dorsally as well as caudally. The ventral aspects of the lungs had areas with moderate consolidation and air bronchograms, particulary of the right side. Intra- and interlobular septa as well as ‚ground-glass'-like opacities, air bronchograms and multiple indentations in the pulmonary pleura were seen caudodorsally on both sides. The precardial mass was hypodense centrally and isodense peripherally relative to the surrounding soft tissues, and there were mineralized areas ventrally. The accumulation of contrast medium after intravenous administration (2 ml/kg body weight, infusion rate 4 ml/sec; Ultravist^®^-300, Schering, Germany) was limited to peripheral areas of the mass and to small nodules between the mass, the heart and the thoracic wall. The thoracic wall appeared normal. Based on the CT examination, a diagnosis of mediastinal or pleural neoplasia and moderate multilobar pneumonia was made. An abscess was unlikely because of the additional nodules identified by the contrast medium.

**Figure 3:**
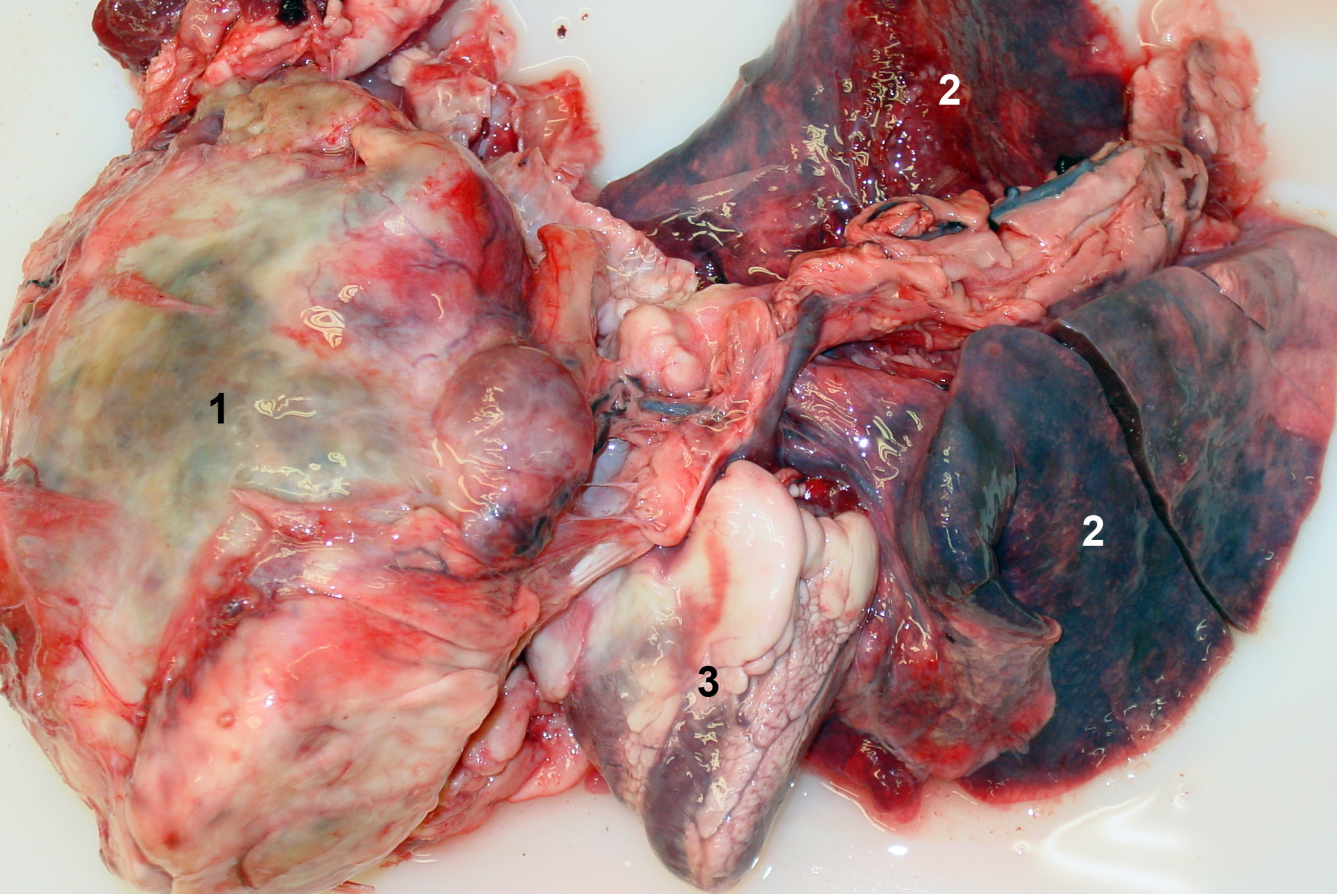
**Gross pathological findings**. Gross pathological findings in a miniature goat with thoracic osteosarcoma. 1 Precardial mass, 2 Lung, 3 Heart.

**Figure 4:**
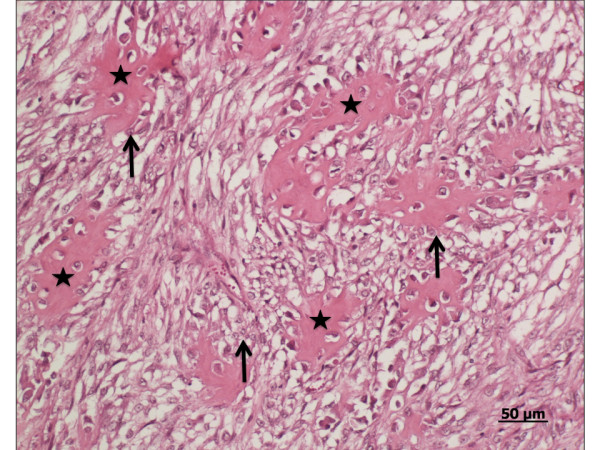
**Histological findings**. Histological section of osteosarcoma in a miniature goat. Irregular osteoid islets (asterisk) are surrounded by malignant osteoblasts (arrow). Some osteoblasts are also seen in the islets. HE staining.

Because the prognosis was grave, the goat was euthanased and a post-mortem examination was carried out. Examination of the thorax revealed a large firm white oval mass cranial to the heart (Figure 3). The mass displaced the heart to the right side of the thorax and resulted in compression of the heart, trachea and lungs. The cut surface of the mass had cystic lesions and a central area of necrosis. Adhesions between the mass and the pericardium and sternum were seen in the cranial thorax, and there were multiple nodules ranging in diameter from 0.5 to1.0 cm in the right pleural space. Several contact metastases, which were not connected directly to the ribs, were observed on the costal pleura. The ribs had no macroscopic lesions. Histological examination of the mass and neoplastic nodules revealed irregular osteoid islets, which were surrounded and occasionally infiltrated by malignant osteoblasts (Figure 4). The neoplastic osteoblasts were oval, elongated or spindle-shaped, and the nuclei were generally pleomorphic and often eccentrically placed. There were a few mitotic figures, and a large necrotic area was seen in the centre of the tumour. The definitive diagnosis was thoracic osteosarcoma with contact metastases on the costal pleura and diaphragm. In addition, there was severe acute pulmonary oedema with severe purulent pneumonia in the ventral lung lobes (presumably caused by aspiration) and mild verminous pneumonia in the diaphragmatic lung lobes.

The lead clinical signs in this patient were bilateral distension of the jugular veins, tachycardia and tachypnoea. Distension of the jugular veins primarily suggests right-sided cardiac insufficiency, but in the absence of cardiac signs, may indicate obstruction of the cranial vena cava by a thrombus or compression of the vein by an abscess, tumour or thoracic effusion [17]. In our patient, right-sided cardiac insufficiency could not be ruled out because of the tachycardia and arrhythmia and the right ventricle appeared enlarged on ultrasonograms. However, a combination of ultrasonography, radiography and CT allowed a tentative diagnosis of a tumour. The CT exam provided detailed images of the thoracic organs, which could not be accomplished with ultrasonography. A recent CT study of the thorax in 26 healthy goats [18] was very helpful for interpreting the lesions in our patient. An abscess was not part of the differential diagnosis because the total leukocyte count and concentrations of fibrinogen and total solids were within the reference ranges. The advanced age of our patient favoured a diagnosis of neoplasia. Differential diagnoses for the thoracic tumour included thymoma, thymic lymphoma [1], metastatic carcinoma and tumour of the aortic bodies. Thymic lymphoma tends to occur in younger animals, whereas thymoma has been reported more often in adult or aged animals [19]. Furthermore, regional lymph nodes, which were normal in our patient, are commonly involved in thymic lymphoma, but not in thymoma. A fine needle aspirate or ultrasound-guided biopsy of the mass was not carried out because of the grave prognosis and advanced age of the patient. Instead, a postmortem examination served to provide a definitive diagnosis. Because there were no indications of skeletal involvement, the osteosarcoma was considered extraskeletal. Extraskeletal osteosarcoma has been described in dogs, primarily in the mammary gland, spleen, gastrointestinal and urogenital tracts, liver, skin and subcutis [10]. However, to our knowledge, this tumour has not been reported in domestic animals. Interestingly, a pleural osteosarcoma with pulmonary involvement was recently described in the human literature [20].

## Conclusion

The differential diagnosis in goats with distended jugular veins, tachycardia and tachypnoea should include abscess and neoplasia in addition to right-sided heart failure. Ultrasonography, radiography and computed tomography are useful for diagnosing neoplasia and determining the extent of the lesions. To our knowledge, this case report is the first description of extraskeletal osteosarcoma of the thorax in goats and serves to broaden the diagnostic spectrum of thoracic disease in this species. Extraskeletal osteosarcoma should be part of the differential diagnosis in goats with thoracic neoplasia.

## Consent

Consent was obtained from the owner of the goat for publication of this case report and any accompanying images.

## Authors' contributions

UB prepared the manuscript and supervised the clinical examination, CS performed the ultrasonographic examination, EF examined the goat, MBB contributed to the clinical research, NP performed the postmortem examination, SO was responsible for the CT examination. All authors have read and approved the manuscript.
